# Exploring recent patterns of migration of doctors to the United Kingdom: a mixed-methods study

**DOI:** 10.1186/s12913-023-10199-y

**Published:** 2023-11-04

**Authors:** N. Brennan, N. Langdon, T. Gale, N. Humphries, A. Knapton, M. Bryce

**Affiliations:** 1 CAMERa, Peninsula Medical School, Faculty of Health, University of Plymouth, Drake Circus, Plymouth, PL4 8AA UK; 2 Graduate School of Healthcare Management, Royal College of Surgeons in Ireland, Dublin, Ireland; 3 Strategic Modelling Analysis and Planning Limited (SMAP), Winchester, UK

**Keywords:** Health workforce research, Health workforce migration, Health workforce retention, Doctors, Mixed-methods, UK

## Abstract

**Introduction:**

A shortage of doctors is currently one of the biggest challenges faced by the healthcare workforce in the United Kingdom (UK). While plans are in place to increase the number of medical school places, in the short-term this gap will need to continue to be filled by the international recruitment of doctors. The aim of this study is to identify key factors that explain the patterns of migration of doctors to the UK, in order to aid the development of policies to recruit and retain a sustainable workforce.

**Methods:**

We analysed General Medical Council (GMC) secondary data on the patterns of migration of internationally trained doctors (2009–2019). Qualitative interviews were conducted with 17 stakeholders by videoconferencing which were audio-recorded, transcribed and thematically analysed using NVivo.

**Results:**

In 2019, 34.5% of UK doctors were trained internationally mainly in India, Pakistan, Italy, Nigeria, Greece, Romania and Egypt. Most new registrations by internationally trained doctors from 2009–2019 did not have a specialty at the time of initial registration (96.2% in 2019). Only a relatively small number of these doctors go on to gain specialist or GP registration (11.6% within 5 years and 27.2% within 10 years of registration). The stakeholder interviews highlighted training opportunities and career progression as the main drivers of migration. The barriers internationally trained doctors face regarding specialty training included differences between UK and destination health systems, systematic bias, bureaucracy and selection processes not being accessible.

**Conclusion:**

This study makes a contribution to the literature by identifying recent patterns in the migration of doctors to the UK. The UK’s dependence on internationally trained doctors has important global implications as source countries are losing skilled health workers which is undermining their health systems. In keeping with the WHO Global Code on the International Recruitment of Healthcare Personnel, policymakers need to consider how to reduce the UK’s reliance on internationally trained doctors, particularly from countries on the safeguard list whilst continuing the drive to increase medical school places. Additional support is required for internationally trained doctors, to ensure that they get on the training programmes they seek, enabling their career progression.

**Supplementary Information:**

The online version contains supplementary material available at 10.1186/s12913-023-10199-y.

## Introduction

A global workforce crisis in healthcare is imminent [1]. The World Health Organisation (WHO) has estimated that by 2030 there will be a shortage of approximately 10 million health workers globally [2] that is 20% of the workforce needed to keep healthcare systems going [1]. In the UK, a shortage of doctors in the National Health Service (NHS) is particularly acute. This shortage has reportedly been driven by years of insufficient investment in training new staff, inadequate workforce planning, and lack of government accountability [3]. In June 2022, the British Medical Association recorded 10,582 vacancies in secondary care medical staff positions across the UK [3]. The Royal College of Physicians census showed that only 52% of consultant vacancies were filled in 2022 [4]. The consequence of this workforce crisis is mounting with the COVID-19 pandemic intensifying these issues even more and is resulting in increased workloads, deteriorating staff wellbeing and poor retention [3].

The NHS has become increasingly reliant on overseas doctors since the 1960’s [5, 6]. Doctors with overseas qualifications are a core part of the UK medical workforce, with over 110,748 (36%) of licensed doctors in the UK in 2021 being internationally trained [7]. Between 2018 and 2019 the number of doctors on the General Medical Council (GMC) Register (including those without a licence to practise) increased by 11,321, with more than 60% of that increase being internationally trained doctors [8]. A recent systematic review of the literature found that the main drivers of the migration of doctors to the UK were employment opportunities, poor working conditions in home country, better training and development opportunities in the UK, desire to work in a different environment, opportunities to gain clinical experience, financial gain, desire for life change and better quality of life [8]. At the same time, an average of 5% of doctors left the UK medical workforce each year from 2013 to 2019; data captured on their reasons for leaving demonstrated that approximately 2.1% (10,624) are going overseas [8]. The most popular places UK-trained doctors migrate to are other high-income English-speaking countries especially Australia and New Zealand [8].

The WHO Global Code on the International Recruitment of Healthcare Personnel (2010) recommends that high income countries should train and retain sufficient doctors to meet demand rather than rely on immigration [2]. Whilst The Global Strategy on Human Resources for Health Workforce 2030 stipulates that countries need to halve their dependence on foreign-trained professionals by 2030. Given the UK’s commitment to the WHO Global Code, the reliance on the international recruitment of doctors to address workforce shortages is not a desired or sustainable solution. This has been recognised in the 2023 NHS Long-Term plan which stipulates that in the long-term the UK needs to train sufficient doctors to meet domestic demand; but it also acknowledged that this will take time as it takes 5 years to train a doctor and several more years for them to specialise, thus the UK will need to ensure that high-skilled doctors are able to join the NHS [9]. It is predicted that in 15 years’ time approximately 9–10.5 per cent of the workforce will be recruited from overseas, compared to almost a quarter now [10].

In order to train sufficient doctors to meet domestic demand the UK will need to increase medical school places. In 2018/2019 there were 42,190 medical students in total [11] with 8,730 being graduates in the UK [12]. It is estimated that medical school places have increased by 30% since 2010 [13]. In order to meet the NHS requirement for sustainability and growth, the Medical Schools Council states that an expansion in the number of medical students will eventually be required to increase places by 5,000 to produce 14,500 medical graduates each year [14]. This would address NHS staffing needs while also ensuring that a reasonable degree of overseas recruitment continues [14].

A recent scoping review [8] of the drivers and barriers of the migration of doctors to the UK identified some studies that have explored the patterns of migration of doctors to the UK. One of these studies examined the pattern of migration of doctors from 2003–2008 using registration data from the GMC [15]. During this time period India, Pakistan and South Africa were the most common source countries. Another study also examined GMC registration data relating to the changes in doctor migration to the UK since the extension of the European Union in May 2004, finding that the majority originated from Commonwealth countries^1^ [16]. However these studies are now out of date and there have been some significant global events since then that may have impacted on the patterns of migration of doctors to the UK, including the UK leaving the European Union in 2016 thus justifying the need to explore more the recent trends in patterns of migration.

Noticeably, both of the aforementioned studies used data from the national register of doctors held by the GMC in their studies [16, 17]. This data is the most comprehensive nationwide data source of the migration of doctors to the United Kingdom [17]. The GMC produces an annual report on the *State of Medical Education and Practice* which presents data on the number of internationally trained doctors joining the UK workforce [8]. Our study provides a more in-depth and longitudinal analysis of GMC registration data focusing on previously unexplored aspects of the data. Furthermore through qualitative interviews with key stakeholders it seeks to explore the reasons for some of the key trends identified. Effective medical workforce planning is dependent upon an understanding of the complex migration patterns of doctors and the drivers behind them. By summarising the knowledge on this topic, the research will contribute to the wider debate on workforce policy and planning. The research will also identify any significant knowledge gaps in the patterns of migration of doctors to the UK. The aim of this paper is to identify key factors that may explain the patterns of migration of doctors to the UK. Firstly, by identifying the patterns of migration to and from the UK using an analysis of secondary data and secondly by drawing on qualitative interviews that potentially explain some of the key patterns.

## Methods

### Study design

In 2021, our research team conducted a large mixed-methods study that aimed to identify the factors that explain recent and longer-term patterns in the migration of doctors to and from the UK. We report key findings from both the quantitative and qualitative parts of the study in this paper. Further detail on the study methods and all of the findings are available in the final report which has been published online by the GMC [8]

### Analysis of secondary data

The main source of data utilised to understand the recent patterns of migration to the UK was GMC data [18]. The GMC publishes aggregate data from 2006 to present on the current distribution of the medical workforce across the four nations of the UK [19] and on the sources of the medical workforce by country of primary medical qualification (PMQ) [18]. We sampled data over a 10-year period from 2009–2019. Raw data from the register dataset containing the characteristics of doctors and details of their location as derived by the GMC were provided by the GMC to the research team in an Excel spreadsheet. The following assumptions were applied to the data and the following limitations need to be acknowledged:

1. To allow comparison with the data reported in the annual State of Medical Education and Practice (SoMEP) report, the analysis looks at registered and licensed doctors as of the SoMEP census date of the 30th June each year. As such, a doctor who entered the register after the 30th June and left before the next 30th June would therefore not be included in this analysis.
2. Furthermore, in line with the approach taken in SoMEP, doctors who have not registered, or have been suspended or erased from the list of Registered Medical Practitioners are excluded from the analysis*.*
3. The data presented consists of cross-sectional snapshots covering the 12 month period and thus individual doctors were not tracked year-on-year

The data were analysed using descriptive statistics, i.e. frequencies, in Excel by an experienced statistician (AK) [20]. For the purposes of the analysis, the source country of doctors has been grouped by income into high (HICs), middle (MICs) and low income (LICs) countries. This is a useful categorisation as it is known that health worker migration is often connected to the economic status of a country. Other studies on health worker migration have also used this categorisation [21, 22]. In some instances we have also referred to European Economic Area (EEA) countries to demonstrate a particular trend.

### Qualitative research interviews

We conducted a series of qualitative interviews with key stakeholders (*n* = 17) between September and November 2020. The purpose of these interviews was to identify key trends in the migration of doctors to and from the UK and to understand the factors that drive doctors to move between countries during their careers (from a stakeholder perspective).

#### Sampling and participant recruitment

A purposive sampling strategy was adopted to recruit key stakeholders from organisations with appropriate expertise and knowledge about the international migration of doctors to the UK, e.g. professional bodies, medical education training organisations, locum agencies and international regulators. The participant organisations were identified via existing links between the GMC and the organisations, as well as via links between the organisations and the research team. An information sheet was sent to all participants. If the participant wanted to take part in the study they needed to complete a consent form prior to the interview taking place. The research occurred during the COVID-19 pandemic however it did not have an influence on the potential respondents.

#### Data collection

The format of the interviews were semi-structured. A topic guide was developed by the research team to address the research questions but also allowing for the conversation to develop based on the individual participant’s particular perspective and expertise. We did not ask participants to respond from an institutional point of view so their responses may be a mixture of institutional and/or personal views. The interviews were carried out by NL and NB via video-conference call between 22/9/2020 and 5/11/2020. All interviews were audio-recorded and then transcribed by a professional transcriber. The transcriber was bound by a confidentiality agreement. All of the transcripts were anonymised and were referred to by a signifier (country and type of organisation). We conducted interviews with 17 participants across 14 organisations (see Additional file 1). Three of the interviews were group interviews involving 2 participants. A written response to the topic guide was provided by one organisation.

#### Data analysis

The data were thematically analysed in NVivo 12 (QSR) using an inductive approach. The first step was to develop a coding framework. The coding framework was developed by three members of the research team (NL, NB and MB). Each member coded four transcripts independently and then developed their own coding framework. They all then met as a group to compare their individual frameworks and agree a final framework. NL then completed the remainder of the coding. NL had regular meetings with the research team to discuss any potential queries or issues or changes to the coding framework.

### Ethical approval

Ethical approval was received from the University of Plymouth Faculty of Health Research Ethics and Integrity Committee and was approved on the 3^rd^ June 2020 (ref no: 19/20–1222).

## Results

### Patterns of migration to the UK

#### No.s of doctors joining the register

From 2009 to 2019 the number of internationally trained doctors joining the GMC register almost doubled (albeit not linearly), increasing from 4,880 to 9,353 (Table 1). Between 2017–2019 the increase has been particularly noticeable with an increase of 61% during this time. Comparing the data from 2009 with 2019, the number of doctors migrating from HICs has remained relatively stable, whereas the number of doctors from MICs has doubled. Overall, the number of doctors migrating from MICs increased from 2,209 in 2009 to 5,827 in 2019 (Table 1). There has also been an increase in the overall number of doctors from LICs increasing from 216 in 2009 to 876 in 2019. The number of new registrants from HICs has fallen from 3,640 (62.5%) in 2013 to 2,650 (28.3%) in 2019 (Table 1).

**Table 1:** Numbers of internationally trained doctors by relative wealth of their country of origin and UK trained doctors joining the GMC register annually 2009–2019

	**2009**	**%**	**2010**	**%**	**2011**	**%**	**2012**	**%**	**2013**	**%**	**2014**	**%**	**2015**	**%**	**2016**	**%**	**2017**	**%**	**2018**	**%**	**2019**	%
**HICs**	2,455	50	3,281	51	3,648	57	3,451	59	3,640	63	3,828	61	3,085	56	2,488	46	2,495	43	2,459	34	2,650	28
**MICs**	2,209	45	2,868	44	2,408	38	2,161	37	1,939	33	2,148	34	2,154	39	2,594	48	2,968	51	4,209	59	**5,827**	62
**LICs**	216	4	304	5	330	5	287	5	242	4	257	4	253	5	295	5	356	6	488	7	**876**	9
**Internationally trained doctor registrations**	4,880	40	6,453	47	6,386	47	5,899	45	5,821	45	6,233	45	5,492	42	5,377	42	5,819	45	7,156	50	9,353	56
**UK registrations**	7,265	60	7281	53	7,181	53	7,254	55	7,150	55	7,550	55	7,472	58	7,494	58	7,210	55	7,142	50	7,234	44
**Total New Registrations**	12,145	100	13,734	100	13,567	100	13,153	100	12,971	100	13,783	100	12,964	100	12,871	100	13,029	100	14,298	100	16,587	100

Source: GMC Data—Doctor Details and Derived Doctor Location datasets

*HICs* Higher income countries

*MICs* Middle income countries

*LICs* Lower income countries

#### Source countries of doctors migrating to the UK

**Fig. 1:**
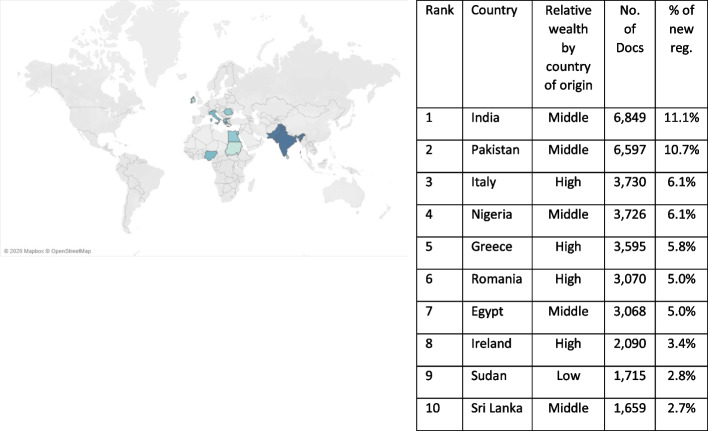
Countries with highest levels of migration to the UK from 2010 to 2019. Source: GMC Data—Doctor Details and Derived Doctor Location datasets

**Fig. 2:**
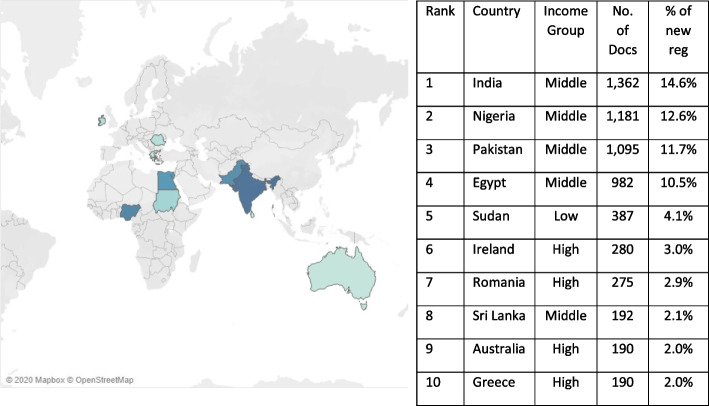
Countries with highest levels of migration to the UK in 2019

The doctors migrating from MICs are primarily coming from India, Pakistan, Nigeria and Egypt (Fig. 1). While the numbers of doctors migrating from these countries dropped in the first half of the decade; between 2015–2019 there was a 328% increase. In 2019, these four countries made up almost half (49.4%) of internationally trained registrations (Fig. 2). The reduction in doctors migrating from HICs during the same time period has been primarily driven by fewer doctors migrating from European countries, with a particularly sharp decrease in internationally trained doctors from Southern European countries, e.g. Italy and Greece, since 2015.

It is important to note that Pakistan, Nigeria and Sudan are on the safeguard list published by the WHO ethical code [23]. Countries on the safeguard list face the most pressing health workforce needs in relation to universal health coverage. The WHO health workforce support and safeguards list 2023 comprises 55 countries.

#### No. of doctors joining specialty and GP register

Most new registrations by internationally trained doctors from 2009–2019 do not have a specialty at the time of initial registration (96.2% in 2019) (Table 2). The trend increased from 10.0% to 15.4% in 2015 and decreased to 3.8% from 2015 onwards. Some doctors could be eligible if they gain equivalence recognition by the relevant royal college. Since 2009, the most common specialties entered were General Practice, Physician and Surgery, however these proportions had lessened by 2019.

**Table 2:** Proportion of internationally trained new registrants joining the specialist and GP register^a^ 2009–2019

	**2009**	**2009**	**2010**	**2010**	**2011**	**2011**	**2012**	**2012**	**2013**	**2013**	**2014**	**2014**	**2015**	**2015**	**2016**	**2016**	**2017**	**2017**	**2018**	**2018**	**2019**	**2019**
Specialist/GP	488	10%	667	10%	809	13%	682	12%	758	13%	788	13%	848	15%	439	8.%	411	8%	316	4%	352	4%
Non-specialist	4,392	90%	5,786	90%	5,577	87%	5,217	88%	5,063	87%	5,445	87%	4,644	85%	4,938	92%	5,408	92%	6,840	90%	9,001	96%

Source: GMC Data—Doctor Details and Derived Doctor Location datasets

^a^The Specialist Register is a list of doctors who have completed specialisation and are eligible to take up appointment in any fixed term, honorary or substantive consultant post in the NHS. The GP Register is a list of doctors who are eligible for appointment as a general practitioner in the UK

Table 3 presents data on internationally trained joiners, excluding those that joined the specialist or GP register at the point of initial registration, who were still registered at the time of analysis. Internationally trained doctors can apply for entry onto the GP/Specialist register at any point via Certificate for Eligibility for Specialist Registration (CESR)/Certificate for Eligibility for GP Registration (CEGPR) [24]. It’s also important to note that those who do go into training could be joining the UK medical training pathway at different points. Table 3 clearly shows that only a small number of non-specialist doctors at registration gain specialist or GP registration in the following years. For example, if we examine the cohort of doctors that registered in 2009, 11.6% (448/3,860 doctors) had entered the specialty register within 5 years, rising to 27.2% for those that have been on the register for 10 years.

**Table 3:** Progression of internationally trained doctors into specialist or GP registration of those not on either register at initial time of registration

	**Year of Specialist or GP registration**												
**Year of registration**	**2010**	**2011**	**2012**	**2013**	**2014**	**2015**	**2016**	**2017**	**2018**	**2019**	**Total**	**Total No. of international trained doctors registered**	**% of GMC registered internationally trained doctors that gained specialisation/became a GP by 2019**
**2009**	**76**	**64**	**65**	**105**	**138**	**125**	**122**	**111**	**115**	**129**	**1,050**	**3,860**	**27.2**
2010	2	93	71	79	132	146	152	146	185	193	**1,199**	5,125	**23.4**
2011	0	2	93	67	89	116	133	139	129	149	**917**	4,856	**18.9**
2012	0	0	0	103	93	77	109	126	130	138	**776**	4,508	**17.2**
2013	0	0	0	0	105	93	87	95	114	138	**632**	4,228	**14.9**
2014	0	0	0	0	1	114	110	88	110	132	**555**	4,566	**12.2**
2015	0	0	0	0	0	1	115	76	79	120	**391**	4,237	**9.2**
2016	0	0	0	0	0	0	1	90	63	90	**244**	4,529	**5.4**
2017	0	0	0	0	0	0	0	1	74	80	**155**	5,052	**3.1**
2018	0	0	0	0	0	0	0	0	0	78	**78**	6,590	**1.2**
2019	0	0	0	0	0	0	0	0	0	0	**0**	8,690	**0.0**

Source: GMC Data—Doctor Details and Derived Doctor Location datasets

### Drivers of migration to the UK & understanding low numbers of internationally trained doctors on specialist register

This section presents key findings from the qualitative data that identify the main factors which attracted doctors to migrate to the UK. It also provides some reasons as to why such a low number of internationally trained doctors join the speciality register.

#### Training opportunities

The opportunity to train in the UK remains a core driver for overseas qualified doctors coming to the UK [25]. Doctors seek postgraduate training opportunities, fellowships, specialty training as well as clinical observerships.

*“There are training opportunities, and structured programmes are good, and the opportunities through training programmes to acquire college diplomas and fellowships is also useful” (Medical Education Training Body #1, UK)*

The stakeholders highlighted the status of UK training on the global stage. For example, the prestige of holding professional qualifications and developed experience from the UK, as well as the transferability of accreditation from the Medical Royal Colleges, which opens doors for career progression due to their reputation and global reach.

*“…it’s useful for people if they want to go back home and practice, it’s external recognition of a quality of attainment, which again it’s a prestige thing I think ”( Medical Education Training Body #1, UK)*

*“[T]hey may well come and seek the opportunity to train in the UK, and that’s attractive either because the professional qualifications and the accreditation transfer ability of royal colleges badges gives people.” (NHS, UK)*

#### Career progression

The opportunity for career progression is another core driver of doctor migration to the UK. We define career progression as completing the different stages of training required to reach consultant level in a particular speciality. Although we acknowledge that career progression can mean different things to different doctors. As the stakeholder above outlined, opportunities exist in the UK for gaining experience, specialisation and career progression. This type of migration tends to occur relatively early on in a doctor’s career so that they can benefit from the postgraduate training opportunities offered within the NHS. This is often compounded by comparatively poor career progression opportunities within one’s home country. Therefore, career progression constitutes a core push and pull factor for internationally trained doctors according to stakeholders.

*“Then the opportunity to then progress your career, either in the UK or somewhere else, so there are a number of people who come and look to migrate early on in their career for that reason.” (NHS, UK)*

*“I think the NHS is attractive because in normal conditions it’s got good learning opportunities for health professionals, and it’s public sector, it provides reasonably fair and ethical treatment for migrant health workers in terms of equal access to career development opportunities and so on” (NGO, UK)*

*“sub-specialisation as well there’s a lot of experience available as well in the UK to do that so it’s quite attractive for career progression” (Recruitment Agency, New Zealand)*

#### Specialist training

Despite the global reputation of UK qualifications, one stakeholder highlighted the need to avoid complacency that internationally trained doctors would continue to seek training opportunities in the UK, when in fact there is a need to offer the same level of support to internationally trained doctors that would be provided to UK trainees.

*“And the offer that we have for people, particularly some of the sort of accreditation and the global recognition, whether it be through research or whether it’s through the royal college set-up, is still held in really high regard, but I do think we need to not be complacent that people should just choose here and then we should hope that they get on and be the best they can be, actually we do have to support people in a way that we support our UK trainees to go through their training programme if we want to see them, well if we want to uphold that reputation, but also if we want people to succeed” (NHS, UK)*

Barriers may include differences between home and destination health systems, for example, differences in the specialties practiced, and *how* those specialties are practiced, which may make transition to the UK difficult. There may also be differences in the qualifications obtained by doctors coming from overseas, which may be to the disadvantage of internationally trained doctors, due to perceived prioritisation of UK trainees.

*“if you’d asked me pre-Covid the barriers to coming here, the only barrier is if you have a postgraduate qualification or a royal college exam qualification you are at the front of the queue, if you have the standard what we call the PLAB, but if you have PLAB you’re at the back of the queue” (Locum Agency #1, UK)*

Perceptions of systematic bias through the prioritisation of UK trainees over overseas doctors, or the idea that overseas doctors are on an unequal footing in the UK system, is a significant concern mentioned by interviewees.

*“I believe the UK selection processes at ST1 are quite biased against non-UK people, they tick all the boxes for equality and diversity but the way that it’s set up is set up deliberately to favour foundation doctors who’ve done their foundation in the UK” (Medical Education Training Body #1, UK)*

The issue described by interviewees, is that selection processes are not accessible to international candidates, but in fact, an implicit language exists that UK trainees understand.

*“it’s like the hidden curriculum in undergraduate medicine, if you know it you can learn it, you can address it, if you’re unaware of it you have no clue how to answer these questions” (Medical Education Training Body #1, UK)*

In addition to potential bias within the health system, overseas doctors also face barriers when it comes to registration in the UK, and advancing on to the specialist register. One interviewee mentioned the high bureaucratic barriers for Australian doctors migrating to the UK, and the need to provide a large evidential requirement, “*almost having to go to find a kindergarten teacher to prove that you really did speak English*” (Regulator, Australia). Three of the interviewees, mentioned the difficulties posed by working with the GMC (Medical Education Training Body #1, UK; Locum Agency #1, UK; Medical Education Training Body #2, UK) when it came to unrecognised PMQs, and the additional hurdle this presents.

*“to actually get onto the specialist register the GMC makes it nothing but a nightmare if you haven’t got a recognised PMQ. So we’ve had an example recently of a doctors we’ve had to have what we call a ‘letter of equivalence’ drawn up from the royal college, and it just becomes a complete hassle, it’s a headache” (Locum Agency #1, UK)*

## Discussion

### Summary of main findings

The aim of our study was two-fold. Firstly, to identify the recent patterns of migration of internationally trained doctors to the UK and secondly, to understand the reasons for these patterns. Our study found, that in 2019, 56% of the doctors who joined the GMC register were internationally trained. From 2009–2019 the number of internationally trained doctors joining the GMC medical register almost doubled. The main source countries are India, Pakistan, Italy, Nigeria, Greece, Romania and Egypt. The vast majority of new registrations by internationally trained doctors do not have a specialty at the initial time of registration. Only a relatively small number of doctors who are non-specialist doctors at registration go on to gain specialist or GP registration (11.6% within 5 years and 27.2% within 10 years). The interviews with stakeholders highlighted the prestige of UK training on the global stage and that the main reasons doctors come to the UK are for training opportunities and for career progression. They also highlighted the barriers internationally trained doctors face regarding specialty training which included differences between UK and destination health systems, systematic bias, bureaucracy and selection processes not being accessible.

### Comparison with existing literature

The data demonstrates the UK’s increasing reliance on internationally trained doctors. This trend is contrary to the WHO global code on the international recruitment of healthcare personnel which states that member states should become self-sufficient in their supply of health-workers [2]. Despite being published in 2010 the UK is still nowhere near self-sufficient in its supply of doctors. It is estimated that medical school places have increased by 30% since 2010 [13] but it is predicted that another 11,000 more places need to be created to address the shortage of doctors by 2030 [26].

The finding that there has been an increase in doctors migrating from MICs in the time period we explored highlights a potential changing trend. While the UK is recruiting quite small numbers of doctors from LICs, there is still an issue of ‘brain drain’ from MICs. ‘Brain drain’ refers to the mass movement of health care workers from low and middle-income countries (LMICs) to high-income countries, resulting in shortages of health care workers in LMICs [27]. There is widespread agreement that health worker migration severely undermines the capacity of LMICs to develop competent health care systems. Our results showed how three of the main source countries (Nigeria, Sudan and Pakistan) were on the safeguarding list. However, despite this code of practice, doctors continue to migrate from these countries.

Although the stakeholders identifying the main driver for doctors migrating to the UK as being for training opportunities and for career progression, very few internationally trained doctors are gaining specialist or GP registration when they get to the UK. Furthermore not many had gone on to join the specialist or GP register within 10 years. For UK trained doctors that enter the medical training pathway it takes a minimum of 5 years to become a GP and up to 10 years to become a consultant. According to the Royal College of Physicians, there is a 25% loss of doctors between medical school and consultant level which implies that 75% achieve consultant status [28]. Our analysis found that only 27.2% of all internationally trained doctors joined the register within 10 years and while this figure includes all internationally trained doctors joining the register, not just those that enter the UK medical training pathway, it implies that around 70% of internationally trained doctors do not get on the specialty register which is concerning. The possible reasons for this trend identified in the qualitative interviews with stakeholders related to differences between UK and destination health systems, systematic bias, bureaucracy and selection processes not being accessible. This leads to a number of concerns. Firstly that doctors come to the UK with expectations for progression that are not met and secondly that these doctors may continue to face hurdles progressing through the UK health system. A study on Ireland found that when the migration motivation of the IMG clashes with the requirements of the health system this leads to dissatisfaction among the IMG medical workforce and onward migration resulting in a cycle of ‘brain gain, waste and drain” [29]. Further research is needed to explore the perspectives of the international trained doctors on the reasons for such a low number gaining specialist/GP registration.

It could be argued that specialty registration is a poor or too blunt proxy measure for training/career progression however other research supports our findings. A systematic review by Khan [30] et al. also found that internationally trained doctors face hurdles in career progression and passing exams. Furthermore a qualitative study conducted in 2015 highlighted the difficulties for internationally trained doctors to enter and then progress through the UK health system [31]. To get ahead doctors relied upon “old school networks”, particularly when trying to attain the top positions. The study also highlighted the difficulties of attaining training positions for internationally trained doctors if not already working within the UK system. Internationally trained doctors can become caught in a “training trap” and are unable to progress which has traditionally been the case for IMGs in Ireland [32]. A study of Polish doctors in Sweden found that specialists were often reduced to novice doctor [33]. In the USA it was found that migrant doctors find it difficult to obtain fellowships and have limited long-term career options [34]. The difficulties experienced by internationally trained doctors progressing within the UK system is concerning, and could be suggestive of institutional discrimination [25].

### Implications for policy-makers

The implications of the study findings for policy-makers is that the UK needs to prioritise and continue its drive to increase medical school places in the UK as well as reduce its reliance on internationally trained doctors, particularly from countries on the safeguard list. In addition, more support is needed for internationally trained doctor’s to ensure any of the additional barriers to career progression and registration on the specialty or GP register are overcome. This could include the development of specific pathways to specialty registration for overseas registrants. Policymakers in LMIC’s should concentrate on enhancing policies to retain more doctors in their countries.

### Strengths & limitations of the research

The strengths of the study are that it was mixed-methods with the qualitative interviews providing a supplementary analysis of the same problem area. The GMC data set was robust. The team was multi-disciplinary with researchers from a variety of backgrounds including research, clinical and a data analyst.

It is important to note that the GMC’s medical register does not equate with the workforce (as some doctors who are registered may not be working as doctors, or may be working in the private sector etc.). It does indicate though how many doctors are eligible to join the UK medical workforce.

The limitations of the study are that it did not include the views or experiences of migrating doctors which should be the focus of future research.

## Conclusions

This study makes a contribution to the literature by identifying recent trends in the patterns of migration of doctors to the UK. In keeping with the WHO Global Code on the International Recruitment of Healthcare Personnel, policymakers need to consider how to reduce the UK’s reliance on internationally trained doctors, particularly from countries on the safeguard list whilst continuing the drive to increase medical school places. The UK’s dependence on internationally trained doctors has important global implications as it means source countries are losing skilled health workers undermining their health systems and which goes against the global solidarity of WHO code. Additional support is required for internationally trained doctors, to ensure that they get on the training programmes they seek, enabling them to fulfil their career aspirations and progress their careers.

### Supplementary Information

**Additional file 1.**

## Data Availability

The datasets generated and/or analyzed during the current study are not publicly available. Data may however be available from the corresponding author upon reasonable request and with permission of the Principal Investigator (Dr. Nicola Brennan, corresponding author), the GMC and the University of Plymouth Faculty of Health Research Ethics and Integrity Committee.

## Footnotes

1 see www.thecommonwealth.org/our-member-countries. for full list of commonwealth countries.

## References

[1] KPMG. Human: Solving the global workforce crisis in healthcare. 2023. https://kpmg.com/xx/en/home/insights/2019/03/human-solving-the-global-workforce-crisis-in-healthcare.html. Accessed 3 Feb 2023.

[2] WHO. WHO Global Code of Practice on the International Recruitment of Health Personnel. 2010. https://www.who.int/publications/i/item/wha68.32. Accessed 24 Apr 2023.

[3] BMA. NHS medical staffing data analysis. 2022. https://www.bma.org.uk/advice-and-support/nhs-deliveryand-workforce/workforce/nhs-medical-staffing-dataanalysis#:~:text=High%20vacancies&text=As%20of%20June%202022%2C%20over,are%20having%20on%20staff%20retention. Accessed 30 Oct 2023.

[4] RCP census finds record number of physician jobs unfilled. https://www.rcplondon.ac.uk/news/rcp-census-finds-recordnumber-physician-jobsunfilled#:~:text=More%20than%20half%20(52%25),Physicians%20and%20Surgeons%20of%20Glasgow. 10.1136/bmj.o1782 35850972

[5] Simpson JM: Migrant architects of the NHS: South Asian doctors and the reinvention of British general practice (1940s–1980s). In: Migrant architects of the NHS. edn.: Manchester University Press; 2018.

[6] Snow S, Jones E: Immigration and the National Health Service: putting history to the forefront. History and Policy 2011.

[7] State of medical education in practice: the workforce report. 2022. www.gmc-uk.org/-/media/documents/workforce-report-2022---full-report_pdf-94540077.pdf.

[8] Brennan N, Langdon N, Bryce M, Gale T, Knapton A, Burns L, Humphries N. Drivers of international migration of doctors to and from the United Kingdom. General Medical Council; 2021. 10.1186/s12960-022-00789-y PMC9927032 36788569

[9] NHS. NHS Longterm Workforce Plan. 2023.

[10] NHS Employers. NHS Long Term Workforce Plan 2023: what employers need to know. 2023.

[11] General Medical Council. State of medical education in practice: the workforce report 2019 and data tables. 2019.

[12] Statista. Number of medical graduates in the United Kingdom (UK) from 2002 to 2021. 2023. https://www.statista.com/statistics/473206/medical-graduates-in-the-united-kingdom-uk/. Accessed 24 Apr 2023.

[13] Harvey A. More medical school places is no cure-all for the UK’s shortage of doctors. 2022. https://www.theguardian.com/society/2022/feb/13/more-medical-school-places-is-no-cure-all-for-the-uks-shortage-of-doctors.

[14] Medical Schools Council. The expansion of medical school numbers in the United Kingdom: Medical Schools Council position paper. 2021.

[15] Young R (2010). Motivations and experience of health professionals who migrate to the United Kingdom. Evaluation of international recruitment of health professionals in England. J Health Serv Res Policy.

[16] Herfs PG (2014). Aspects of medical migration with particular reference to the United Kingdom and the Netherlands. Hum Resour Health.

[17] Young R (2011). A major destination country: the United Kingdom and its changing recruitment policies. Health professional mobility and health systems Evidence from.

[18] General Medical Council. GMC Register Data: Sources of the medical workforce by country. 2019. https://data.gmc-uk.org/gmcdata/home/#/reports/The%20Register/World%20maps/report. Accessed 20 Nov 2019.

[19] General Medical Council. GMC Register Data: Distribution of medical workforce by country. 2019. https://data.gmc-uk.org/gmcdata/home/#/reports/The%20Register/UK%20maps/report. Accessed 20 Nov 2019.

[20] The World Bank. World Bank Country and Lending Groups. 2021. https://datahelpdesk.worldbank.org/knowledgebase/articles/906519.

[21] Williams GA, Jacob G, Rakovac I, Scotter C, Wismar M (2020). Health professional mobility in the WHO European Region and the WHO Global Code of Practice: data from the joint OECD/EUROSTAT/WHO-Europe questionnaire. Eur J Public Health.

[22] Clarke N, Crowe S, Humphries N, Conroy R, O'Hare S, Kavanagh P, Brugha R (2017). Factors influencing trainee doctor emigration in a high income country: a mixed methods study. Hum Resour Health.

[23] WHO. WHO health workforce support and safeguards list 2023. 2023. https://www.who.int/publications/i/item/9789240069787. 10.2471/BLT.23.290191 PMC10225944 37265683

[24] General Medical Council. Certificate of Eligibility for Specialist Registration or Certificate of Eligibility for GP Registration application. 2023. https://www.gmc-uk.org/registration-and-licensing/join-the-register/registrationapplications/specialist-application-guides/specialist-registration-cesr-or-cegpr. Accessed 15 Sep 2023.

[25] Davda LS, Gallagher JE, Radford DR (2018). Migration motives and integration of international human resources of health in the United Kingdom: Systematic review and meta-synthesis of qualitative studies using framework analysis. Hum Resour Health.

[26] Torjesen I (2022). England needs 11 000 more medical student places a year, say doctors’ leaders. BMJ.

[27] Oshotse C: An Ethical Analysis of the Global Medical Brain Drain. Voices Bioethics 2019, 5.

[28] Royal College of Physicians. Double or quits: calculating how many more medical students we need. 2018. https://www.rcplondon.ac.uk/news/double-or-quits-calculating-how-many-more-medical-students-we-need. Accessed 11 Sep 2023.

[29] Humphries N, Tyrrell E, McAleese S, Bidwell P, Thomas S, Normand C, Brugha R (2013). A cycle of brain gain, waste and drain - a qualitative study of non-EU migrant doctors in Ireland. Hum Resour Health.

[30] Khan F, Chikkatagaiah S, Shafiullah M, Nasiri M, Saraf A, Sehgal T, Rana A, Tadros G, Kingston K (2015). International Medical Graduates (IMGs) in the UK—a systematic review of their acculturation and adaptation. Int Migration Integ.

[31] Legido-Quigley H, Saliba V, McKee M (2015). Exploring the experiences of EU qualified doctors working in the United Kingdom: a qualitative study. Health Policy.

[32] Humphries N, Bidwell P, Tyrrell E, Brugha R, Thomas S, Normand C: “I am kind of in stalemate”. The experiences of non-EU migrant doctors in Ireland. Health professional mobility in a changing Europe 2014:233.

[33] Wolanik Boström K, Öhlander M: A troubled elite? Stories about migration and establishing professionalism as a Polish doctor in Sweden. 2012.

[34] Reichert SF, Thomas AK, Humphrey H. Mentoring a vulnerable cohort for success: the special case of the foreign medical graduate. In: Mentoring in academic medicine. edn. Philadelphia: ACP Press; 2010.

