# Supplementary material for: Exploring recent patterns of migration of doctors to the United Kingdom: a mixed-methods study
Source: BMC Health Serv Res. 2023 Nov 4;23:1204. doi: 10.1186/s12913-023-10199-y (PMC10625180; doi:10.1186/s12913-023-10199-y)
Supplement: Supplementary file 1 — Additional file 1. [file 12913_2023_10199_MOESM1_ESM.docx]

Appendix 1: List of organisations interviewed

|  | **Country** | **Type of Organisation** | **No of interviewees** |
| --- | --- | --- | --- |
| 1. | UK | NHS | 1 |
| 2. | UK | Med Ed/training | 1 |
| 3. | UK | Med Ed/training | 1 |
| 4. | UK | Professional | 1 |
| 5. | UK | Professional | 1 |
| 6. | Australia | Regulator | 1 |
| 7. | Canada | Professional | 1 |
| 8. | New Zealand | Higher Education | 1 |
| 9. | USA | Regulator | 1 |
| 10. | Ireland | Regulator | 2 |
| 11. | UK | Locum Agency | 1 |
| 12. | UK | Locum Agency | 1 |
| 13. | UK | Research Group | 2 |
| 14. | UK | Recruitment Agency | 2 |
| 15. | UK | Trade Union | Written response |
| Totals | 10 UK 1 Ireland 1 Australia 1 New Zealand 1 Canada 1 USA | 15 organisations | 17 interviewees  1 written response |
